# Evaluating the risk of osteopenia-related adverse events with antiepileptic drugs: a pharmacovigilance study based on the FAERS database

**DOI:** 10.3389/fphar.2025.1685289

**Published:** 2025-10-13

**Authors:** Nan Yang, Xiang Han, Hongning Hua, Yumeng Wang, Yulin Chen, Yue Zhou, Haoyu Feng

**Affiliations:** ^1^ Third Hospital of Shanxi Medical University, Shanxi Bethune Hospital, Shanxi Academy of Medical Sciences, Tongji Shanxi Hospital, Taiyuan, China; ^2^ Xinqiao Hospital ARMY Medical University, Chongqing, China

**Keywords:** antiepileptic drugs, osteopenia, phenytoin, valproic acid, gabapentin, eslicarbazepine

## Abstract

**Background:**

Controversy remains regarding the association between antiepileptic drugs and osteopenia, and this study aims to investigate and address this gap using real-world data.

**Materials and methods:**

This study included data from the United States (U.S.) Food and Drug Administration Adverse Event Reporting System (FAERS) from the first quarter of 2005 to the first quarter of 2025. A disproportionate analysis method and Bonferroni-corrected p-value were used to detect association signals between antiepileptic drugs and osteopenia. Additionally, subgroup analyses were conducted to explore differences among different age and gender groups.

**Results:**

Among the 206,680 adverse events associated with 12 commonly used antiepileptic drugs recorded in the FAERS database during the study period, 181 were attributable to osteopenia. Positive drug safety signals were detected for phenytoin, valproic acid, gabapentin, and eslicarbazepine.

**Conclusion:**

Previous studies have only identified a link between phenytoin and osteopenia, while the relationship between valproic acid and this adverse event remains controversial, and gabapentin and eslicarbazepine have not been systematically reported. The findings provide the first evidence of an association between these four antiepileptic drugs and osteopenia, offering insights and guidance for clinical recognition and prevention of such events.

## 1 Introduction

Epilepsy is a chronic neurological disorder caused by various etiologies, characterized by recurrent, episodic, and transient dysfunction of the central nervous system due to excessive synchronized discharge of brain neurons, with significant neurobiological and sociopsychological implications (Manford, 2017). Its pathogenesis is multifactorial, involving ion channel abnormalities, neurotransmitter imbalances, and neural network reorganization, with genetic factors and acquired brain injury jointly contributing to the pathological process (Shorvon, 2011). Clinical manifestations are diverse, including loss of consciousness and tonic-clonic seizures during generalized tonic-clonic seizures, as well as focal seizures accompanied by localized sensory and motor abnormalities. The severity of this disease manifests in multidimensional harm. Specifically, recurrent seizures significantly increase the risk of accidental injury or death, progressive cognitive decline impairs patients’ social adaptation abilities, and social stigma and disease-related shame further exacerbate psychological disorders. In patients with severe disease, the risk of suicide is further amplified (Giambarberi and Munger Clary, 2022). As a major global public health issue, approximately one in ten people will experience an epileptic seizure in their lifetime. Specifically, there are currently about 65 million epilepsy patients worldwide, with one in 26 people affected in the United States, placing a heavy burden on families and healthcare systems (Milligan, 2021).

In this context, antiepileptic drugs have irreplaceable core value in clinical application. They exert therapeutic effects by regulating neuronal excitability, inhibiting abnormal discharges, or stabilizing neuronal cell membranes. Their clinical application should be selected based on seizure type, patient age, and tolerability (Ovsiew, 2004; Liu et al., 2017). First-line drugs are the clinical first choice. Carbamazepine is highly effective for partial seizures and secondary generalized seizures, acting by blocking sodium channels to inhibit high-frequency neuronal discharges. Additionally, ethosuximide is the first-line drug for absence seizures, acting on T-type calcium channels. Phenytoin controls tonic-clonic seizures by stabilizing cell membranes to reduce sodium ion influx. Valproic acid is a broad-spectrum antiepileptic drug suitable for various seizure types, with mechanisms involving enhancing gamma-aminobutyric acid (GABA) activity and inhibiting sodium and calcium channels (Kanner and Bicchi, 2022). Additionally, second-line drugs are often used as adjunctive therapy or alternative options. Gabapentin aids in the treatment of partial seizures by binding to voltage-dependent calcium channels. Lamotrigine combines sodium channel blockade with glutamate release inhibition, making it suitable for both partial and generalized seizures. Levetiracetam reduces abnormal discharges by regulating synaptic vesicle protein 2A and has a high safety profile. Additionally, oxcarbazepine, a derivative of carbamazepine, has better tolerability. Topiramate and zonisamide assist in controlling refractory epilepsy through multiple mechanisms. Finally, third-line drugs are used for refractory cases. Eslicarbazepine enhances control of partial seizures through sodium channel blockade, while lacosamide promotes sodium channel inactivation and is suitable for patients who do not respond well to other drugs. Clinical drug use should follow the principle of individualization, prioritizing monotherapy with first-line drugs, and gradually adding second- and third-line drugs when efficacy is inadequate to optimize treatment outcomes. It is noteworthy that a variety of clinically commonly used antiepileptic drugs (AED), such as valproate, carbamazepine, lamotrigine, and pregabalin, not only belong to the first-line agents for epilepsy treatment but are also widely applied in the management of multiple psychiatric disorders in clinical practice. Meanwhile, a comorbid relationship between some psychiatric disorders and epilepsy is frequently observed in clinical settings. This expansion of the clinical application scope refers specifically to the long-term administration of these AED to a broader population beyond patients with epilepsy, and such an expansion may further exacerbate the risk of adverse events associated with long-term drug use (Bosak et al., 2015).

Osteopenia is a metabolic bone disorder characterized by reduced bone mass and impaired bone microarchitecture, though it does not meet the diagnostic criteria for osteoporosis, it poses significant clinical risks. Its pathophysiological mechanisms involve impaired osteoblast function and enhanced osteoclast activity, leading to an imbalance between bone formation and resorption, ultimately resulting in a decrease in bone density of 1–2.5 standard deviations below peak bone mass (Karaguzel and Holick, 2010). As a precursor to osteoporosis, osteopenia progresses insidiously, often without obvious symptoms, yet significantly increases the risk of fractures, particularly fragility fractures in the hip and spine. When occurring in specific populations, this risk is greatly elevated, leading to chronic pain, limited mobility, and even disability. Studies show that the fracture risk for patients with osteopenia is 2–3 times higher than that of individuals with normal bone mass, and this risk increases sharply with age. After menopause, women experience accelerated osteopenia due to decreased estrogen levels, making them more prone to progressing from osteopenia to osteoporosis (Gopinath, 2023). Additionally, osteopenia is associated with various chronic diseases, such as diabetes and hyperthyroidism, which further exacerbate osteopenia by affecting calcium and phosphorus metabolism. If left untreated, osteopenia becomes irreversible, significantly increasing the difficulty of subsequent treatment and the burden on healthcare resources. Therefore, early identification and intervention are crucial for halting disease progression and reducing fracture risk (Reid and McClung, 2024).

Given the widespread clinical use of antiepileptic drugs and the long-term nature of their treatment regimens, drug-related adverse events associated with these medications are increasingly drawing attention (Perlman et al., 2019). Specifically, existing studies have reported that such drugs may increase the risk of adverse events related to osteopenia (Pack and Morrell, 2001). Among these, as a first-line drug for treating epilepsy, the association between phenytoin and osteopenia has been widely reported and has become a mainstream consensus (Kanner and Bicchi, 2022). However, the link between valproic acid, another first-line drug for treating epilepsy, and osteopenia remains controversial (Min et al., 2020; Tao et al., 2021; Wang et al., 2025). Additionally, the association between other antiepileptic drugs and osteopenia adverse events has not been systematically reported. Despite these research gaps, potential associations may exist between these antiepileptic drugs and osteopenia-related adverse events. As a quarterly updated database maintained by the United States (U.S.) Food and Drug Administration (FDA), the Adverse Event Reporting System (FAERS) is one of the largest publicly available databases on drug adverse reactions, providing researchers with raw data from the FDA’s official website. FAERS is managed by the FDA and integrates adverse event (AE) reports submitted by healthcare professionals, patients, pharmaceutical companies, and other stakeholders, serving as a data source for retrospective observational pharmacovigilance studies. It can detect trends in unexpected adverse events that may not have been identified due to limitations in clinical trial participants. Therefore, this study, based on the FAERS database, aims to investigate the potential association between commonly used antiepileptic drugs and osteopenia, with the goal of providing valuable reference information for rational drug use in clinical practice.

## 2 Materials and methods

### 2.1 Data sources

As noted in the Introduction, this study focused on clinically commonly used antiepileptic drugs, which included 4 first-line agents, 6 second-line agents, and 2 third-line agents. This study selected data from FAERS covering the first quarter of 2005 to the first quarter of 2025 involving 12 antiepileptic drugs for pharmacovigilance research (https://www.fda.gov). Specifically, the search was conducted on August 12, 2025. The 12 antiepileptic drugs included in the database search corresponded to the following names: CARBAMAZEPINE, ETHOSUXIMIDE, PHENYTOIN, VALPROIC ACID, GABAPENTIN, LAMOTRIGINE, LEVETIRACETAM, OXCARBAZEPINE, TOPIRAMATE, ZONISAMIDE, ESLICARBAZEPINE and LACOSAMIDE. The relevant content includes seven subset files: patient demographics and management information (DEMO), drug information (DRUG), adverse event coding (REAC), patient outcomes (OUTC), report source (RPSR), treatment start and end dates for the reported drug (THER), and drug indication (INDI). Following FDA recommendations, these files are linked and sorted with related adverse events using PRIMARYID, CASEID, and drug_seq, establishing a standardized linking method. Additionally, the International Conference on Harmonization (ICH) guidelines for international safety reporting are used to dynamically adjust the data, thereby constructing a standardized reference system and optimizing the overall effectiveness of the data adaptation process.

### 2.2 Procedures

The Medical Dictionary for Regulatory Activities (MedDRA) Version 26.1 establishes a clear hierarchical classification system for medical terminology, including preferred terms (PT) for signs and symptoms, higher-level terms (HLT), higher-level groups term (HLGT), and system organ classes (SOC) (Brown et al., 1999). Among these, the MedDRA terminology adverse reaction classification criteria for PT categorize drugs into four types: PS (primary suspicion), SS (secondary suspicion), C (concomitant effect), and I (interaction). To enhance the accuracy of results, this study will focus on analyzing reports where the drug’s role_cod is “PS” (primary suspicion). At the same time, the study used MeSH subject headings to query the generic names and brand names of 12 epilepsy drugs to ensure completeness. In addition, the research conducted in-depth cleaning of the original data, screening and removing reports with record errors, missing key information, and redundant duplicates, with the aim of minimizing the possibility of certain events being overrepresented in the analysis and thereby enhancing the value of data mining. Following FDA recommendations, if the PRIMARYID is the same, the latest FDA_DT is selected as the time identifier; if both FDA_DT and CASEID are the same, the higher PRIMARYID is chosen to eliminate duplicate reports submitted by different individuals or organizations.

### 2.3 Statistical analysis

This study employs disproportionate analysis as a signal detection method in pharmacovigilance analysis. Specifically, in the definition and identification of adverse events (AE), four algorithms—reporting odds ratio (ROR), proportional reporting ratio (PRR), information content (IC), and empirical Bayesian geometric mean (EBGM)—are comprehensively utilized to conduct disproportionate analysis (Supplementary Material). To identify all potential drug safety risks, reports meeting the positive criteria of any one of the four methods are classified as positive adverse event reports. Furthermore, ROR is recognized to effectively reduce bias interference, estimate relative risk more accurately, and serve as a more robust analytical method. Thus, it was adopted as the primary analytical approach for presentation in this study (Rothman et al., 2004). Additionally, to reduce Type I errors caused by multiple comparisons, the calculated p-value is adjusted using the Bonferroni method to further enhance the accuracy and reliability of the study results. Based on this, the study focused on further analysis of the drugs that exhibited positive signals. In terms of gender grouping, participants were divided into male and female groups; in terms of age stratification, recipients under 60 years of age were defined as the younger group, and recipients aged 60 years and above were defined as the older group. Finally, to ensure the efficiency, accuracy, and reproducibility of the analysis process, this study utilized R 4.4.2 and its integrated development environment RStudio.

## 3 Results

### 3.1 Descriptive analysis

**FIGURE 1:**
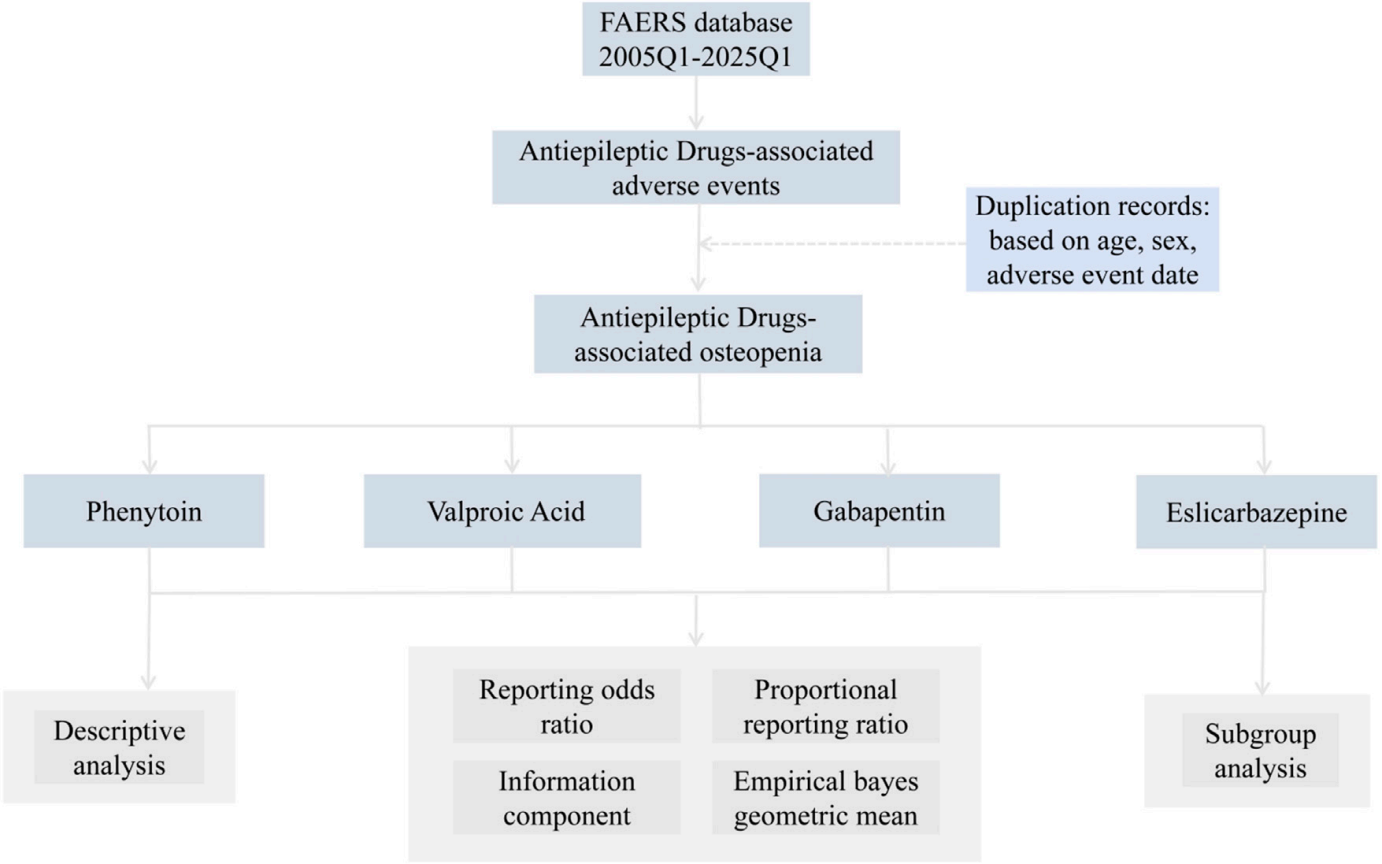
Flow chart showing the analysis process of the study.

**FIGURE 2:**
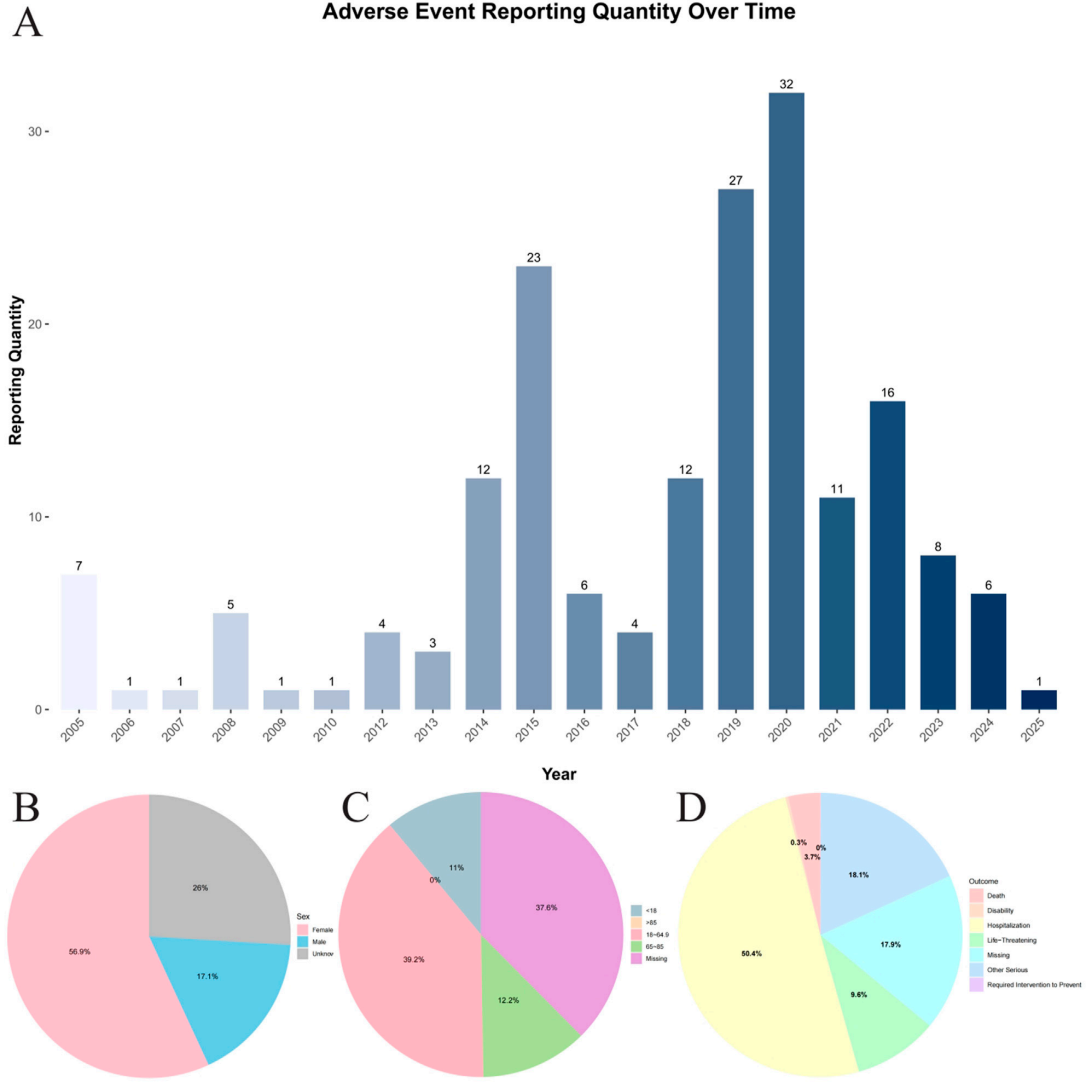
Total Summary of Baseline Information. **(A)** Annual distribution of reported cases of osteopenia associated with antiepileptic Drugs in the FAERS database (2005Q1–2025Q1). **(B)** Distribution of reported cases by gender group. **(C)** Distribution of reported cases by age group. **(D)** Distribution of reported cases by outcome group.

Between the first quarter of 2005 and the first quarter of 2025, the FAERS database recorded a total of 206,680 reports on commonly used epilepsy medications (Figure 1; Table 1). This includes first-line medications such as carbamazepine (n = 15,199), ethosuximide (n = 478), phenytoin (n = 7,844), and valproic acid (n = 8,149), second-line medications such as gabapentin (n = 54,126), lamotrigine (n = 33,937), levetiracetam (n = 47,801), oxcarbazepine (n = 6,159), topiramate (n = 17,567), and zonisamide (n = 2,451), as well as third-line medications such as eslicarbazepine (n = 59) and lacosamide (n = 13,617). Among these, 181 cases were associated with osteopenia events (Figure 2). Among these, the highest reported frequency was for gabapentin (n = 60), while the lowest number of osteopenia events was associated with eslicarbazepine (n = 2). In all adverse event reports related to antiepileptic drugs, the proportion of female patients (n = 103,439) was higher than that of male patients (n = 68,245). When examining the age distribution of patients taking antiepileptic drugs, the younger age group had a higher number of patients than the older age group, with the largest number of patients (79,203) falling within the 18–65 age range. Furthermore, after applying a disproportionate analysis algorithm in this study, the monitoring results showed that phenytoin, valproic acid, gabapentin, and eslicarbazepine had statistically significant positive associations.

**TABLE 1:** Clinical distribution characteristics of adverse event reports associated with antiepileptic drugs.

Characteristics	Antiepileptic drugs (N, %)	Phenytoin (N, %)	Valproic acid (N, %)	Gabapentin (N, %)	Eslicarbazepine (N, %)
Number of Events	206,680	7,844	8,149	54,126	59
Sex, n%					
Female	103,439 (50.0%)	3,407 (43.4%)	3,339 (41.0%)	28,322 (52.3%)	32 (54.2%)
Male	68,245 (33.0%)	3,560 (45.4%)	3,616 (44.4%)	16,348 (30.2%)	26 (44.1%)
Unknown	34,996 (16.9%)	877 (11.2%)	1,194 (14.7%)	9,456 (17.5%)	1 (1.7%)
Age, n%					
<18	20,170 (9.8%)	652 (8.3%)	1,500 (18.4%)	925 (1.7%)	26 (44.1%)
18–64.9	79,203 (38.3%)	3,566 (45.5%)	3,671 (45.0%)	21,986 (40.6%)	20 (33.9%)
65–85	25,543 (12.4%)	1,446 (18.4%)	688 (8.4%)	11,009 (20.3%)	3 (5.1%)
>85	2,909 (1.4%)	121 (1.5%)	67 (0.8%)	1,178 (2.2%)	0 (0%)
Unknow	78,855 (38.2%)	2059 (26.2%)	2,223 (27.3%)	19,028 (35.2%)	10 (16.9%)
Outcome					
CA	4,623 (2.2%)	66 (0.8%)	305 (3.7%)	153 (0.3%)	0 (0%)
DE	13,679 (6.6%)	399 (5.1%)	751 (9.2%)	4,556 (8.4%)	0 (0%)
DS	3,233 (1.6%)	73 (0.9%)	106 (1.3%)	1,128 (2.1%)	0 (0%)
HO	40,973 (19.8%)	1,683 (21.5%)	2,336 (28.7%)	7,095 (13.1%)	8 (13.6%)
LT	7,909 (3.8%)	228 (2.9%)	439 (5.4%)	1,295 (2.4%)	1 (1.7%)
OT	66,230 (32.0%)	2,252 (28.7%)	2,682 (32.9%)	11,664 (21.5%)	16 (27.1%)
RI	171 (0.1%)	5 (0.1%)	5 (0.1%)	31 (0.1%)	0 (0%)
Unknown	69,862 (33.8%)	3,138 (40.0%)	1,525 (18.7%)	28,204 (52.1%)	34 (57.6%)

Abbreviations: CA, Congenital Anomaly; DE, Death; DS, Disability; HO, Hospitalization-Initial or Prolonged; LT, Life-Threatening; OT, Other Serious Important Medical Event; RI, Required Intervention to Prevent Permanent.

### 3.2 Signal mining

**FIGURE 3:**
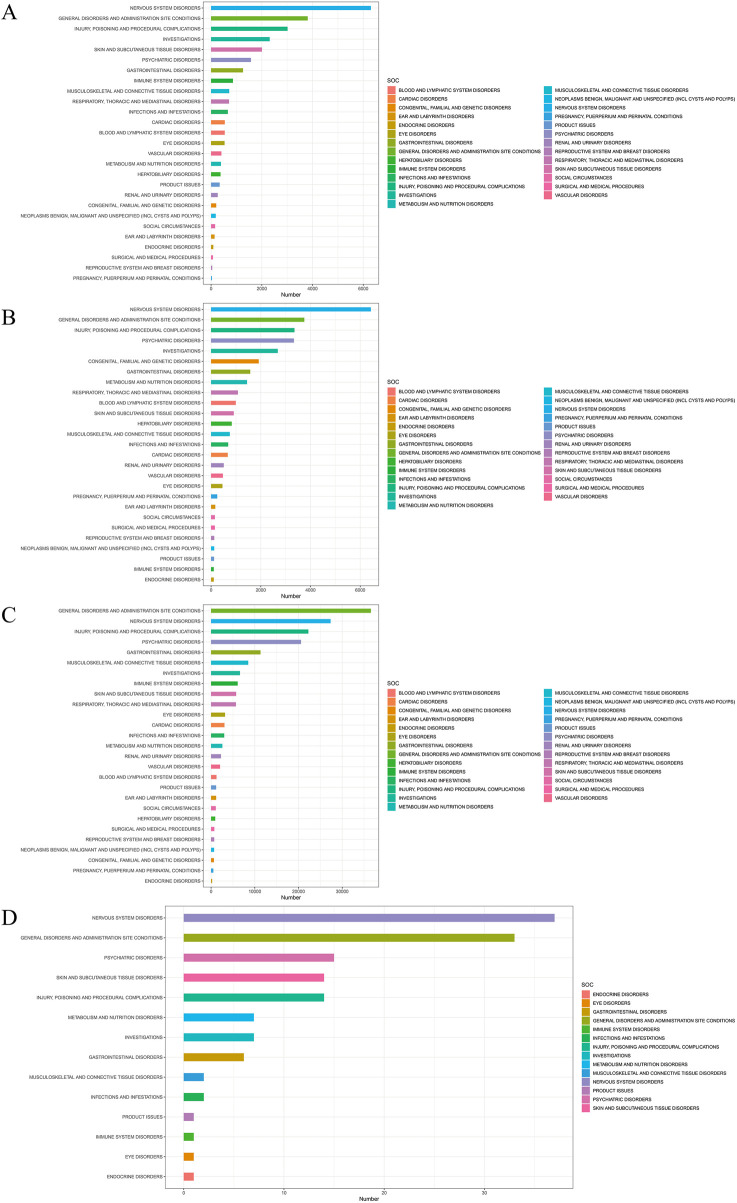
Signal detection of four antiepileptic drugs with positive significance at the system-organ classification (SOC) level. The results are presented using a bar chart. The height of the bar chart represents the quantity. **(A)** Signal detection of Phenytoin at the SOC level. **(B)** Signal detection of Valproic Acid at the SOC level. **(C)** Signal detection of Gabapentin at the SOC level. **(D)** Signal detection of Eslicarbazepine at the SOC level.

**FIGURE 4:**
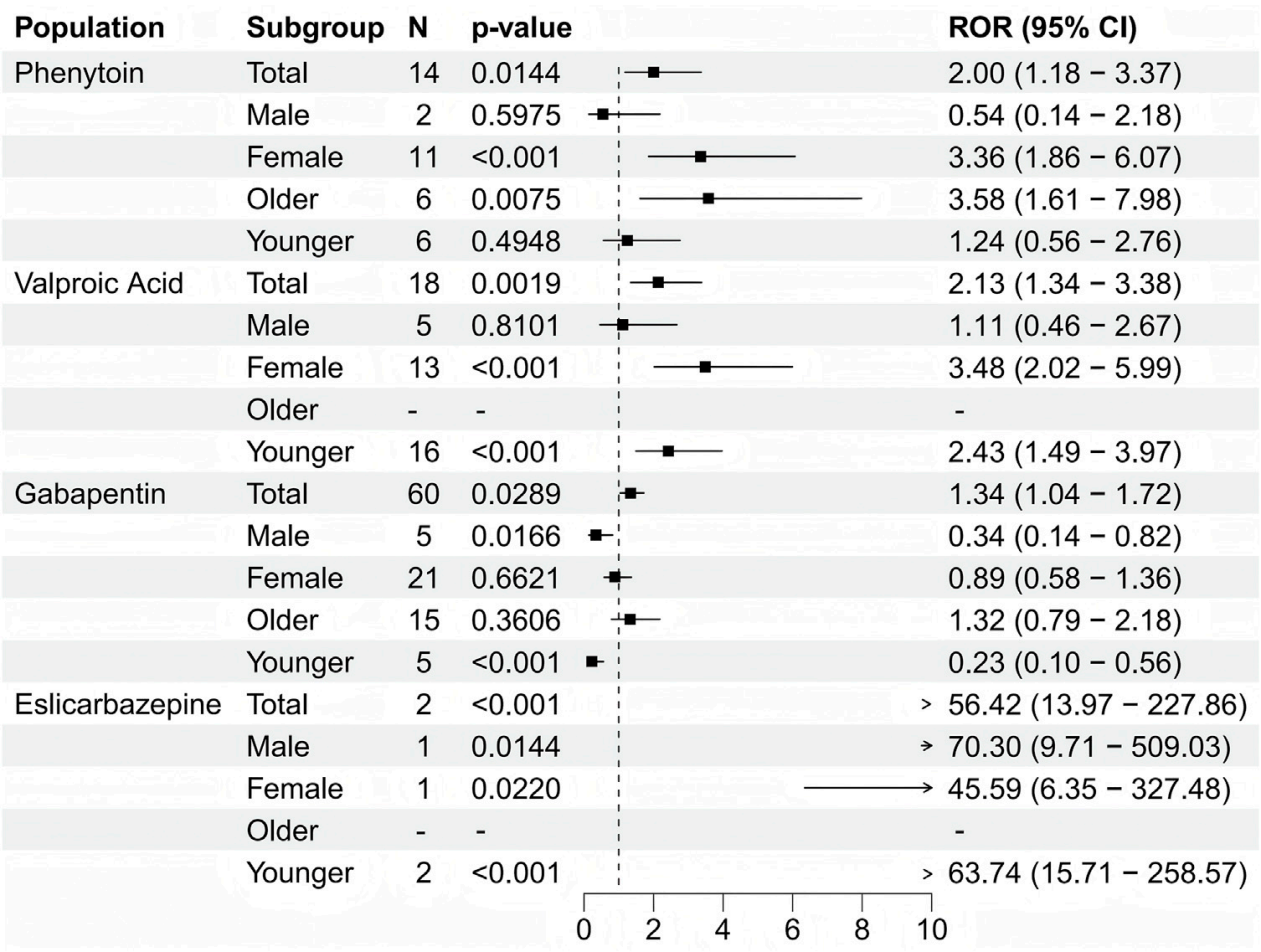
Forest plot showing the results of four antiepileptic drugs with positive significance that tested after disproportionate analysis.

When focusing on the SOC level, a total of 27 organ systems were affected by these four antiepileptic drugs that showed positive signals. Specifically, NERVOUS SYSTEM DISORDERS and GENERAL DISORDERS AND ADMINISTRATION SITE CONDITIONS accounted for the highest proportions (Figure 3). It's worth noting that, when focusing on the PT level related to osteoporosis, the following medications were identified: Phenytoin (ROR (95% CI) = 2 (1.18–3.37)), Valproic Acid (ROR (95% CI) = 2.13 (1.34–3.38)), gabapentin (ROR (95% CI) = 1.34 (1.04–1.72)), and eslicarbazepine (ROR (95% CI) = 56.42 (13.97–227.86)) showed positive signals in the results of the four differential analyses (Figure 4; Table 2). Additionally, the sample size for eslicarbazepine, which showed the highest signal, was small (n = 2), which may introduce bias in subsequent analyses. Furthermore, the research conducted subgroup stratification analyses for the four antiepileptic drugs that showed positive overall dimensional signals. The results of the subgroup analysis indicated that in the phenytoin group, the signal remained positive in the female subgroup and the elderly subgroup, while the signal turned negative in the male subgroup and the young subgroup. However, the results of the pharmacovigilance analysis for valproic acid showed different results. Specifically, while the female group and the young group were positive, the male and elderly groups were negative. Additionally, in the analyses of gabapentin and eslicarbazepine, this research found significant data gaps, resulting in no meaningful outcomes.

**TABLE 2:** Subgroup analysis of antiepileptic drugs based on age and gender.

	Subgroup	N	ROR (95%Cl)	PRR (χ^2^)	EBGM(EBGM05)	IC(IC025)	p-value
Phenytoin	Total	14	2 (1.18–3.37)	2 (6.95)	1.99 (1.18)	1 (0.16)	0.014
	Male	2	0.54 (0.14–2.18)	0.54 (0.76)	0.54 (0.14)	−0.88 (-2.31)	0.597
	Female	11	3.36 (1.86–6.07)	3.36 (18.19)	3.35 (1.86)	1.75 (0.65)	0.001
	Older	6	3.58 (1.61–7.98)	3.58 (11.15)	3.58 (1.61)	1.84 (0.29)	0.007
	Younger	6	1.24 (0.56–2.76)	1.24 (0.28)	1.24 (0.56)	0.31 (-0.83)	0.495
Valproic acid	Total	18	2.13 (1.34–3.38)	2.13 (10.77)	2.13 (1.34)	1.09 (0.34)	0.002
	Male	5	1.11 (0.46–2.67)	1.11 (0.06)	1.11 (0.46)	0.15 (-1.05)	0.810
	Female	13	3.48 (2.02–5.99)	3.48 (22.9)	3.47 (2.01)	1.8 (0.79)	0.001
	Older	0	–	–	–	–	–
	Younger	16	2.43 (1.49–3.97)	2.43 (13.42)	2.43 (1.48)	1.28 (0.46)	0.001
Gabapentin	Total	60	1.34 (1.04–1.72)	1.34 (5.11)	1.34 (1.04)	0.42 (0.04)	0.029
	Male	5	0.34 (0.14–0.82)	0.34 (6.38)	0.34 (0.14)	−1.55 (-2.56)	0.017
	Female	21	0.89 (0.58–1.36)	0.89 (0.29)	0.89 (0.58)	−0.17 (-0.78)	0.662
	Older	15	1.32 (0.79–2.18)	1.32 (1.13)	1.31 (0.79)	0.39 (-0.36)	0.361
	Younger	5	0.23 (0.1–0.56)	0.23 (12.66)	0.23 (0.1)	−2.1 (-3.08)	0.001
Eslicarbazepine	Total	2	56.42 (13.97–227.86)	55.63 (107.31)	55.62 (13.77)	5.8 (-0.15)	0.001
	Male	1	70.3 (9.71–509.03)	68.94 (66.97)	68.93 (9.52)	6.11 (-1.1)	0.014
	Female	1	45.59 (6.35–327.48)	45.06 (43.09)	45.06 (6.27)	5.49 (-1.1)	0.022
	Older	0	–	–	–	–	–
	Younger	2	63.74 (15.71–258.57)	62.47 (120.98)	62.45 (15.4)	5.96 (-0.15)	0.001

Abbreviations: ROR, Report Odds Ratio; PRR, Proportional Reporting Ratio; IC, Information Component; EBGM, Empirical Bayesian Geometric Mean and P value, Adjusted P value.

## 4 Discussion

This study investigates 12 of the most commonly used antiepileptic drugs in clinical practice, aiming to provide a more comprehensive and precise monitoring of the potential risk of osteopenia associated with these medications during clinical use. This offers meaningful Supplementary Material for patients in making medication choices. The findings indicate that certain antiepileptic drugs used in the population are associated with osteopenia. Among these, phenytoin, valproic acid, gabapentin, and eslicarbazepine were found to have a significant association with osteopenia. These findings address gaps in current research on the skeletal effects of these drugs and reveal previously unidentified risks of osteopenia associated with gabapentin and eslicarbazepine. It is worth noting that the association between the aforementioned four drugs and osteopenia is significant, while the association with other antiepileptic drugs is weaker or not significant. This difference is the result of multiple mechanisms and factors acting together.

First, the significant impact on the liver enzyme system (especially on vitamin D metabolism) is crucial. Among these, potent liver enzyme inducers such as phenytoin and eslicarbazepine significantly upregulate the cytochrome P450 enzyme system, accelerating the catabolic metabolism of vitamin D and thereby reducing the levels of active vitamin D in circulation, which affects calcium absorption (Zhou, 2008; Schoretsanitis et al., 2022). In contrast, although carbamazepine and oxcarbazepine are also enzyme inducers, their induction levels are lower compared to phenytoin (Cohen et al., 2024). Additionally, this inconsistency or lack of significance may also stem from study heterogeneity, limited evidence accumulation, or short clinical data periods. Beyond vitamin D metabolism, direct and indirect effects on bone cells also play a significant role. Specifically, phenytoin inhibits osteoblast proliferation and differentiation and may also stimulate osteoclast activity (Nakade et al., 1996). Valproic acid, as a histone deacetylase inhibitor, interferes with osteoblast differentiation, leading to reduced bone formation (Fan et al., 2019; Diemar et al., 2020). As for gabapentin, which is minimally metabolized by the liver and has no significant enzyme-inducing effects, it may influence bone metabolism through indirect pathways, including calcium channel regulation or neurotransmitter-related regulation (Reyes Fernandez et al., 2022). Finally, eslicarbazepine may also exert similar direct or indirect effects on bone cells (Hirsch et al., 2023). Furthermore, the use of AED may also induce changes in bone composition through other mechanisms, including impairing intestinal calcium absorption, inhibiting the cellular response to parathyroid hormone, triggering hyperparathyroidism, and causing calcitonin deficiency (Pack and Morrell, 2001; Pack, 2003; Ali et al., 2004). In summary, the disruption of calcium homeostasis caused by these effects on bone cells may further exacerbate osteopenia. In addition to the above considerations, drug exposure patterns and study-related factors may also influence these outcomes. Specifically, as long-established medications with extensive long-term usage data, phenytoin, valproic acid, and gabapentin are more likely to be associated with osteopenia, which is a progressive condition that may become apparent after prolonged exposure. In contrast, long-term bone density data for newer antiepileptic drugs (such as lamotrigine, levetiracetam, and lacosamide) are limited, and their weaker associations may reflect shorter exposure durations or smaller effects on metabolic pathways (Wang et al., 2024). Baseline bone health, lifestyle factors, comorbidities, and other confounding variables in the epilepsy patient population, as well as potential publication bias toward positive associations, further exacerbate the observed differences. Additionally, valproate has been associated with Fanconi syndrome, which suggests that valproate may induce renal tubular dysfunction. This dysfunction, in turn, leads to increased urinary excretion of calcium and phosphorus, ultimately resulting in the development of osteopenia (Hawkins and Brewer, 1993; Lande et al., 1993). In summary, the significant association between phenytoin, valproic acid, gabapentin, and eslicarbazepine and osteopenia is the result of the combined effects of vitamin D metabolism interference, direct and indirect abnormalities in bone cell function, disruption of calcium homeostasis, and cumulative exposure effects. The weaker association with other antiepileptic drugs is due to a combination of factors, including differences in mechanisms, limited long-term data, and study heterogeneity.

Among commonly used antiepileptic drugs, phenytoin, valproic acid, gabapentin, and eslicarbazepine are significantly associated with osteopenia, while other antiepileptic drugs show weaker or no significant association. This may be partly attributed to their unique pharmacokinetic and pharmacodynamic interactions with concomitant medications, which may also interfere with bone metabolism (Johannessen Landmark and Patsalos, 2010; Zaccara and Perucca, 2014). Among these, phenytoin is a potent inducer of CYP3A4 and CYP2C9. When co-administered with liver enzyme inducers such as rifampicin, it exacerbates the catabolism of vitamin D, synergistically reducing the levels of 1,25-dihydroxyvitamin D in circulation and impairing intestinal calcium absorption (Niemi et al., 2003). Eslicarbazepine may interact with glucocorticoids through mild induction of CYP3A4, although its enzyme-inducing potency is relatively weak, it may still interfere with the activation pathway of vitamin D. Gabapentin is primarily excreted in its unchanged form via the kidneys. When co-administered with nephrotoxic drugs (e.g., nonsteroidal anti-inflammatory drugs, aminoglycoside antibiotics), nephrotoxicity is enhanced, impairing renal 1α-hydroxylase activity, which is critical for vitamin D activation (He et al., 2024). Additionally, co-administration with tetracycline antibiotics or loop diuretics further exacerbates this interaction, worsening calcium homeostasis imbalance. Valproic acid interferes with vitamin K metabolism by inhibiting CYP2C9. When co-administered with warfarin, it may impair bone mineralization, potentially exacerbating osteoporosis (Wieringa et al., 2024). In summary, these drug interactions mediated by changes in enzyme activity, renal function, calcium metabolism regulation, and hormonal control exacerbate the interference of phenytoin, valproic acid, gabapentin, and eslicarbazepine with bone metabolism, making them more significantly associated with osteopenia compared to other antiepileptic drugs that interact less or have a lesser impact on these pathways.

The results of the gender subgroup analysis indicate that higher signal intensity was observed in the female subgroup. This is attributed to a combination of female-specific physiological characteristics, hormonal dynamics, fragile bone metabolism, and drug-related interactions. Estrogen, as a key regulator of bone homeostasis, exerts its effects by inhibiting osteoclast activity and enhancing osteoblast function. However, estrogen levels in women fluctuate significantly, making their bone metabolism particularly sensitive to drug-induced interference (Li et al., 2020). Phenytoin accelerates estrogen metabolism by inducing CYP450 enzymes, while valproic acid may inhibit aromatase, and the two drugs synergistically exacerbate estrogen deficiency (Kastrati et al., 2011; Moawad et al., 2023). This effect is more pronounced in postmenopausal women who already have insufficient endogenous estrogen. Additionally, women have lower peak bone mass and a higher proportion of trabecular bone. Combined with anatomical characteristics, along with often lower baseline levels of vitamin D and calcium due to dietary patterns or insufficient sunlight exposure, this may amplify the effects of drug-mediated metabolic disorders, leading to secondary hyperparathyroidism and accelerated bone resorption (Fitzpatrick et al., 2024). Furthermore, pharmacokinetic interactions further exacerbate the risk. Women are more likely to use sex hormone drugs or drugs that affect bone metabolism in combination, and these drugs may have synergistic effects with the aforementioned four antiepileptic drugs. Specifically, the activation of hepatic drug enzymes induced by phenytoin reduces the efficacy of progestogens, while the nephrotoxic potential of gabapentin is enhanced by the use of nonsteroidal anti-inflammatory drugs, thereby impairing renal calcium reabsorption (Niemi et al., 2003; He et al., 2024). Additionally, behavioral factors and more intensive clinical monitoring (such as more frequent bone density screening in postmenopausal women) may exacerbate observed gender differences by increasing detection sensitivity. In summary, estrogen-dependent vulnerability, intrinsic bone characteristics, drug interactions, and behavioral dynamics contribute to the higher susceptibility of women to osteopenia associated with these four antiepileptic drugs.

In studies involving different age subgroups, the research indicates that the elderly subgroup in the phenytoin sodium population exhibits higher positive signal values, while the drug safety analysis results for valproic acid show that the younger population is positive. This differential pattern may be attributed to the combined effects of drug-specific mechanisms, age-dependent bone metabolic characteristics, physiological reserves, and clinical exposure features. Phenytoin primarily accelerates vitamin D metabolism through potent induction of CYP450 enzymes, which may exacerbate the age-related characteristic of increased bone resorption in the elderly (Chandra and Rajawat, 2021). Older adults inherently have reduced vitamin D synthesis capacity due to decreased sun exposure and skin aging, impaired renal 1α-hydroxylase activity, and increased skeletal sensitivity to parathyroid hormone, making them uniquely susceptible to calcium homeostasis disruption caused by phenytoin. Furthermore, this susceptibility may be further exacerbated by age-related decline in liver and kidney function and comorbidities, which collectively accelerate osteopenia. In contrast, valproic acid exerts its osteotoxic effects by inhibiting histone deacetylase and aromatase, mechanisms that may have a more significant impact on younger individuals during the critical period of peak bone accumulation. Given their active osteoblast activity, young individuals may exhibit higher sensitivity to valproic acid’s inhibition of osteogenesis. Additionally, valproic acid is commonly used in adolescents and young adults, contrasting with phenytoin’s long-term maintenance therapy in the elderly (Tomson, 2000; Ovsiew, 2004). In summary, these dynamic changes reflect a “mechanism-age matching” phenomenon, where phenytoin’s acceleration of bone resorption targets the elderly’s bone structures prone to resorption, while valproic acid’s inhibition of bone formation disrupts the bone mass accumulation mechanisms in young individuals, leading to the observed opposite signal patterns.

This study has several advantages. First, these findings address gaps in current research on the skeletal effects of such drugs and reveal the risk of osteopenia associated with gabapentin and eslicarbazepine, which had not been systematically identified in previous studies. Additionally, compared to other research methods, the continuous analysis based on the FAERS database offers significant advantages in identifying potential safety signals at an early stage. Furthermore, the use of a disproportionate analysis method to monitor new drug safety signals helps enhance healthcare professionals’ and patients’ awareness of the potential risks associated with bisphosphonate drugs, encouraging them to exercise greater caution when prescribing or using these medications, and facilitating the implementation of preventive measures to control the occurrence of such events. Furthermore, by delving into the mechanisms of action and influencing factors of these adverse events, this study will also provide new insights and directions for related scientific research and drug development, further elucidating the mechanisms of action and safety issues of drugs, and providing scientific basis for the development of safer and more effective drugs. Inevitably, this study also has certain limitations. First, the FAERS database is a voluntary reporting system, which inherently introduces selection bias. The adverse event reports it contains may be delayed, incomplete, or inaccurate. This reporting bias is a significant issue that may distort perceptions of drug safety by overemphasizing widely publicized or severe events. Additionally, due to the lack of relevant information in the FAERS database, it is challenging to control for confounding factors such as age, comorbidities, or other factors that may influence health outcomes. Furthermore, underreporting is a recognized limitation, complicating the estimation of incidence rates. Proportional hazards analysis only demonstrates a statistical association between drugs and adverse events, without quantifying risk or establishing a definitive causal relationship. Finally, baseline bone health, lifestyle, comorbidities, and other confounding factors in the epilepsy patient population, as well as potential publication bias toward positive associations, further exacerbate the observed differences. Therefore, higher-quality, larger-scale prospective studies are still needed to confirm the causal relationship and enhance the credibility of current conclusions.

## 5 Conclusion

Utilizing a multidimensional analytical approach on data extracted from FAERS database, the study demonstrated an association between phenytoin, valproic acid, gabapentin, eslicarbazepine and the osteopenia events. This outcome contributes to a deeper clinical insight into the safety spectrum of antiepileptic medications and offers actionable references for formulating early osteopenia prevention protocols. Nevertheless the causal relationship between the studied AED and osteopenia remains unconfirmed and warrants further rigorous research to elucidate.

## Data Availability

Publicly available datasets were analyzed in this study. This data can be found here: Publicly available datasets were analyzed in this study. These data can be found at the FAERS database (https://www.fda.gov).
