# Supplementary material for: Evaluating the risk of osteopenia-related adverse events with antiepileptic drugs: a pharmacovigilance study based on the FAERS database
Source: Front Pharmacol. 2025 Oct 13;16:1685289. doi: 10.3389/fphar.2025.1685289 (PMC12554713; doi:10.3389/fphar.2025.1685289)
Supplement: Supplementary file 1 [file Supplementaryfile1.docx]

**Table S1** The formula to calculate ROR(95%CI)，PRR(χ²)，EBGM(EBGM05) and IC(IC025).

| **Name of Algorithm** | **Formula** | **Positive Signal Criteria** |
| --- | --- | --- |
| **ROR** | ROR=ad/bc | Lower Limit of 95%CI>1, N≥3 |
|  | 95%CI=e ^ln(ROR)±1.96(1/a+1/b+1/c+1/d)^0.5^ |  |
| **PRR** | PRR=(a(c+d))/(c(a+b)) | PRR≥2, X²≥4, N≥3 |
|  | *χ2*=[(ad-bc)^2](a+b+c+d)/[(a+b)(c+d)(a+c)(b+d)] |  |
| **BCPNN** | IC=log_2_a(a+b+c+d)(a+c)(a+b) | IC_025_>0 |
|  | IC_025_=e ^ln(IC)-1.96(1/a+1/b+1/c+1/d)^0.5^ |  |
| **MGPS** | EBGM=a(a+b+c+d)/((a+c)/(a+b)) | EBGM05>2, N>0 |
|  | EBGM05=e ^ln(EBGM)-1.64(1/a+1/b+1/c+1/d)^0.5^ |  |

*ROR*, reporting odds ratio; *PRR*, proportional reporting ratio; *EBGM*, empirical Bayes geometric mean; *IC*, information component; *CI*, confidence interval; *95% CI*, two-sided for ROR; *χ2*, chi-squared; *EBGM05 and IC025*, lower one-sided for EBGM and IC, respectively.
